# The relationship between Chinese college students’ mate preferences and their parents’ education level

**DOI:** 10.3389/fpsyg.2022.907315

**Published:** 2022-11-01

**Authors:** Wuji Lin, Jie Wang, Yutong Liu, Zhuoyu Li, Jingyuan Lin

**Affiliations:** ^1^Institute of Brain and Psychological Science, Sichuan Normal University, Chengdu, Sichuan, China; ^2^Mental Health Education and Guidance Center, Shanxi Normal University, Linfen, Shanxi, China; ^3^School of Psychology, South China Normal University, Guangzhou, Guangdong, China; ^4^School of Psychology, Shenzhen University, Shenzhen, Guangdong, China

**Keywords:** gender role, mate preference, education level, parents, androgyny

## Abstract

Parents have an influence on the formation of their children’s mate preferences. This research conducted two studies to test the relationship between parents’ education level and the gender role characteristics (masculinity and femininity) of ideal mate for college students, and the moderating role of urban-rural residence on this relationship. In study 1, 1,033 participants (627 females) reported their explicit attitude toward gender role characteristics for an ideal mate *via* the Chinese Sex Role Inventory-50. In study 2, we recruited 130 participants (66 females) and used an implicit association test to measure their implicit attitude. Regression-based analyses showed that the higher education level of parents was significantly associated with female students’ mate preferences with high-femininity but low-masculinity traits. For male students, the higher education level of parents was associated with their explicit (not implicit) preferences of mates with high-masculinity but low-femininity traits. The significant moderating effect of urban-rural residence was observed in explicit preference, with the different patterns in gender groups. In conclusion, parents with higher educational attainment might bring up children who are more likely to embrace a partner with non-traditional gender roles (e.g., androgynous individuals, feminine men or masculine women).

## Introduction

According to an evolutionary perspective, the sex differences in personality traits are caused by the different evolutionary pressures faced by males and females (Furnham, 2009; Schwarz and Hassebrauck, 2012; Buss, 2013). In our evolutionary past, hunting (done primarily by males) required skills related to tracking and killing animals, whereas gathering (done primarily by females) required skills related to locating and recalling food sources among an array of vegetation (Wood and Eagly, 2002). Ancestral men and women who were successful in these domains provided more resources to themselves and their offspring and thus gained a reproductive advantage (Balliet et al., 2011).

The differences between males and females are not only physical but also psychological (Lin et al., 2021). In the psychological domain, everyone has two clusters of independent and orthogonal masculine and feminine traits, which are called gender roles. According to this model, gender roles can be classified into four categories: masculine (high in masculinity and low in femininity); feminine (high in femininity and low in masculinity); androgynous (high in both masculinity and femininity); and undifferentiated (low in both masculinity and femininity). These categories are often determined based on the median of score distributions on masculinity and femininity scales (Spence et al., 1975; Bem, 1979; Spence and Helmreich, 1981). Recent research has shown that people in traditional (congruent) gender roles (masculine men and feminine women, Morrison and Shaffer, 2003) are no longer the majority of the population. These results were consistent with previous studies (Juster et al., 2016; Lin et al., 2020). For example, in a study by Lin et al. (2020), 242 (38.72%), 211 (33.76%), 92 (14.72%), and 80 (12.8%) participants were included in androgynous, undifferentiated, masculine and feminine identities, and respectively. It is well-known that gender/sex-binary has been extensively questioned and studied (Hyde et al., 2019; Morgenroth and Ryan, 2021).

Not only have gender roles changed, but mate preference has also changed accordingly. A study among Chinese college students found that men prefer feminine women, which is consistent with tradition, but women prefer masculine and androgynous men, which is partially inconsistent with tradition (Huang, 2013). Another study found that the ideal mate characteristics most valued by Chinese female college students were gentleness and considerateness (Fang et al., 2009), which are feminine characteristics according to traditional views.

Although all of the above studies found that gender roles and mate preference have changed, these changes are only evident in reports of explicit attitudes. Implicit attitudes provide another source of information about mate preferences. An implicit attitude exists outside conscious awareness. Huang (2013) found that the preference for androgyny accounted for a large proportion of the results based on explicit attitudes, but implicit attitudes about gender roles were more consistent with traditional roles based on the individual’s own biological sex. Moreover, the explicit and implicit attitudes about the gender roles of ideal mates were uncorrelated. Additionally, in mate preference, implicit preference was only related to masculine traits. Thus, although people’s expressed preferences have changed over time, a preference for traditional gender roles (masculine men and feminine women) is still evident in implicit attitudes.

Many factors can influence mate preference, among which education is one of the most important (Doosje et al., 1999; Haandrikman and Hutter, 2012). For example, Haandrikman and Hutter (2012) found that the less educated participants indicated that they did not care about the education level of their partners, whereas the more educated participants indicated that the difference should not be too large. However, previous studies only focused on the education level of respondents and disregarded the influence of their parents’ education level. The individual’s own education level cannot fully determine the preference for mate selection because this preference can be traced back to childhood or even earlier (Bereczkei et al., 2002).

Experiences in the family during early childhood have been found to have an impact on sexual preferences (Kim and Smith, 1998; Bereczkei and Csanaky, 2001; Bereczkei et al., 2002). According to attachment theory, parental influence plays a crucial role in children’s future mate choice (Shaver and Mikulincer, 2002). The evolutionary theory of socialization suggests that different types of family environment affect the interactions between family members, which can shape children’s early emotional and behavioral development as well as their future sexual behavior, mate selection strategies, relationship with a partner, and parenting style (Belsky et al., 1991). All of the above theories maintain that parents can play an important role in the formation of their children’s mate preference.

There are significant differences in the early family experiences of children whose parents have different educational levels. Children whose parents have a low level of education are more likely to have greater conflict and less positive communication within the family, and less warmth in parent–child relationships (Chen et al., 2002). Thus, the close association between children’s family experiences and mate preferences, and the influence of parent education on these family experiences, suggests that parent education may predict mate preferences.

Additionally, there is an indirect correlation between the parents’ education level and their children’s mate preference. Several studies have found that the higher the parents’ education level is, the healthier the children’s mental health is (Bao et al., 2016; Qu et al., 2017). Psychological factors such as mental health and personality can also influence mate choice (Giebel et al., 2015; Liu and Ilmarinen, 2019). Second, some studies have found that parenting style and the parent–child relationship are closely related to the parents’ education level (Nilsen et al., 2020; Schrijner and Smits, 2020). Childrearing patterns and the parent–child relationship are also significantly correlated with the children’s mate preferences (Hynie et al., 2006; Buunk et al., 2012; Apostolou, 2015). These studies also suggest that the parents’ education level may be related to mate preference.

The relationship between the parents’ education level and children’s mate preference may be affected by other factors as well. Differences have been found in the mate preferences of people in urban and rural areas (Schvaneveldt and Hubler, 2012; Prakash and Singh, 2014). Schvaneveldt and Hubler (2012) found that rural people were more inclined toward traditional types in terms of mate choice than urban people were, so a difference may also exist in the preference of mates’ gender roles. In addition, some studies have found significant differences between urban and rural areas in parenting style, parent–child relationship quality, and home environment (Zhang et al., 2015, 2020), and these family factors can affect mate preference (Hynie et al., 2006; Buunk et al., 2012; Apostolou, 2015), which suggests that the urban-rural variable can indirectly influence mate preference. Hence, to get a more comprehensive picture of the relationship between college students’ mate preference and their parents’ education level, we tested the area of residence (urban or rural) as a moderator of the association between parental education and their child’s mate preferences.

**Figure 1:**
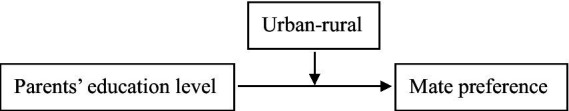
Moderation effect of urban-rural living area on the association between parents’ education level and university students’ mate preference.

In Study 1, a mate preference questionnaire was used to investigate whether there was a correlation between the participants’ explicit mate preference and their parents’ education level. In addition, urban-rural area was tested as a moderator of this correlation (Figure 1).

In Study 2, a single category implicit association test (SC-IAT) was adopted to explore the relationship between participants’ implicit mate preference and their parents’ education level. The Implicit Association Test (Greenwald et al., 1998) can well avoid the influence of individual consciousness and accurately reflect the implicit attitudes. The IAT has been applied to various studies of implicit attitude (see review by Hofmann et al., 2005). We analyzed the relationship between mate preference and parents’ education level and tested whether the results varied for implicit and explicit measures of mate preference based on gender roles.

Some research found that fathers and mothers played different roles on their children’s mate preferences (Apostolou, 2015). In addition, the educational level of the father and mother also led to different parenting styles (Nilsen et al., 2020; Schrijner and Smits, 2020). It might further influence children’s mate preferences. So, we aimed to analyze father’s and mother’s education level separately. We hypothesized that the educational levels of fathers and mothers might be significantly associated with their children’s mate preferences for specific gender role types.

This study explored the relationship between parents’ education level and gender role in mate preference. Further, we explore the differences between implicit and explicit preferences.

## Study 1 explicit mate preference

### Methods

#### Participants

An effect size of *r* = 0.3 was used to conduct a power analysis with G*power 3.1 (Faul et al., 2007). The estimated sample size was no less than 82 to achieve 80% power to detect effects given an α level of 0.05. A total of 1,033 Chinese heterosexual college students volunteered to participate in the study (females = 627, age range = 18–26 years, mean age = 20.642).

#### Measures

##### Gender role—Self

The Sex Role Inventory for Chinese College Students (CSPI-50; Liu et al., 2011) is a self-report questionnaire used to assess an individual’s gender role. The 50-item scale includes three subscales: Masculinity, Femininity, and Neutral. The 16-item Masculinity Subscale includes four factors: leadership, masculinity, rationality, and generosity. The 16-item Femininity Subscale includes three factors: empathy, femininity, thrift, and prudence. The Neutral Subscale includes 18 items that are characteristic of both males and females (e.g., happy, optimistic); these items were not scored. The Cronbach’s alpha coefficients of the Masculinity and Femininity subscales were 0.89 and 0.84, respectively. The demonstrated internal consistency reliability was satisfactory.

##### Gender role—Ideal partner

The CSPI-50 was also adapted for purposes of this study to assess participants’ descriptions of their ideal spouse. The items of the questionnaire were consistent with those of gender role—self. Participants were asked to choose the sex role characteristics of an ideal partner, but not the partner that they were currently dating.

##### Parent education

Parents’ education was a continuous variable represented by years of education (primary school = 6; junior high school = 9; senior high school = 12; associate degree = 15; bachelor’s degree = 16; master’s degree = 19). Mothers’ education and fathers’ education were both used in the analyses.

##### Urban-rural residence

Information about urban-rural was obtained from the question (“Where have you lived most of your life until the age of 18?”) in the questionnaire. It is determined by national administration divisions. It was obtained by participants’ self-report.

#### Procedure

The participants were informed that the survey was anonymous and voluntary. The CSPI-50 was administered online to measure gender role and mate preference. We sent out questionnaire links to participants’ recruitment WeChat groups through a data collection platform^1^ and received 1,204 questionnaires. Questionnaires that were completed in less than 1 min or with the same option for all the questions were considered invalid and excluded, resulting in 1,033 valid questionnaires (85.8% of the original sample). Each participant was remunerated with CNY2.0 upon the completion of their participation.

#### Analysis

First, the descriptive statistics and Pearson’s correlations among the study variables were calculated using SPSS 24.0. Second, we used the SPSS macro PROCESS (Hayes, 2013) to examine the significance of the moderating effect of living area (urban-rural) on the relationship between parental education level and mate preference. The bias-corrected 95% confidence interval (95% *CI*) around each moderation effect was generated using a bootstrap with 5,000 iterations. If the 95% *CI* does not include zero, the effect is considered significant (Preacher and Hayes, 2008). All statistical tests were evaluated at the *p <* 0.05 significance level and constituted two-tailed tests.

### Results

#### Characteristics of participants

The characteristics of the participants are reported in Table 1.

**Table 1:** Characteristics of participants.

		*n*	*Mean*	*SD*
Age				
		1,033	20.64	1.95
Gender				
	Male	406		
	Female	627		
Urban-rural residence				
	Rural	487		
	Urban	546		
Education level				
	Father	1,033	9.76	3.54
	Mother	1,033	8.35	3.94
Male gender role scores				
	Masculinity-ideal partner	406	4.84	0.83
	Femininity-ideal partner	406	5.39	0.89
	Masculinity-self	406	4.81	0.89
	Femininity-self	406	4.80	0.80
Female gender role scores				
	Masculinity-ideal partner	627	5.57	0.81
	Femininity-ideal partner	627	5.03	0.71
	Masculinity-self	627	4.54	0.81
	Femininity-self	627	4.97	0.77

#### Gender role distribution

For descriptive purposes, the participants were categorized based on a median split of the Masculinity and Femininity subscale scores on the CSPI-50, consistent with other research (Spence et al., 1975; Spence and Helmreich, 1981; Lin et al., 2020). The median masculinity and femininity scores were 4.69 and 4.94 in the present study, respectively. The gender roles were classified into four categories: Undifferentiated: *M* < 4.69, *F* < 4.94; Feminine: *M* < 4.69, *F* ≥ 4.94; Masculine: *M* ≥ 4.69, *F* < 4.94; and Androgynous: *M* ≥ 4.69, *F* ≥ 4.94. The gender role distribution is presented in Table 2.

**Table 2:** Gender role distribution (%).

	Masculine	Feminine	Androgynous	Undifferentiated
Males	110 (27.1)	52 (12.8)	139 (34.2)	105 (25.9)
Females	111 (17.7)	161 (25.7)	176 (28.1)	179 (28.5)
Total	221 (21.4)	213 (20.6)	315 (30.5)	284 (27.5)

#### Mate preference distribution

We used the same method to classify mate preferences. They were categorized based on a median split of the gender role scores of mate preferences. The median masculinity and femininity scores were 5.31 and 5.19, respectively. The mate preference distribution is presented in Table 3.

**Table 3:** Mate preference distribution (%).

	Masculine	Feminine	Androgynous	Undifferentiated
Males	30 (7.4)	158 (38.9)	91 (22.4)	127 (31.3)
Females	200 (31.9)	52 (8.3)	217 (34.6)	158 (25.2)
Total	230 (22.3)	210 (20.3)	308 (29.8)	285 (27.6)

#### Correlations among parental education level, gender role, and mate preference

Pearson’s correlations among parents’ education level, gender role scores and mate preference scores are presented in Table 4. For male students, fathers’ education level was positively correlated with the masculinity of an ideal mate (*r* = 0.106, *p* = 0.033) and negatively correlated with the femininity of an ideal spouse (*r* = −0.128, *p* = 0.010); mothers’ education level was positively correlated with the masculinity of an ideal spouse (*r* = 0.105, *p* = 0.034); urban-rural was positively correlated with the parents’ education level (*r* = 0.187, *p <* 0.001; *r* = 0.149, *p* = 0.003). For female students, fathers’ education was positively correlated with the students’ own masculinity score (*r* = 0.103, *p* = 0.010); mothers’ education level was also positively correlated with students’ own masculinity score (*r* = 0.082, *p* = 0.040), and was negatively correlated with the masculinity of an ideal spouse (*r* = −0.111, *p* = 0.005); urban-rural was positively correlated with the parents’ education level (*r* = 0.378, *p <* 0.001; *r* = 0.417, *p <* 0.001) and own masculinity score (*r* = 0.158, *p <* 0.001).

**Table 4:** Correlations between education and for explicit mate preference.

	Variable	1	2	3	4	5	6	7
Males	1.Father’s education level	1						
	2. Mother’s education level	0.570***	1					
	3.Ideal spouse’s masculinity	0.106*	0.105*	1				
	4. Ideal spouse’s femininity	−0.128**	−0.015	0.418***	1			
	5.Own masculinity	0.034	0.031	0.574***	0.412***	1		
	6. Own femininity	−0.052	−0.015	0.455***	0.495***	0.534***	1	
	7. Urban-rural	0.187***	0.149**	−0.031	0.081	0.076	0.028	1
Females	1.Father’s education level	1						
	2. Mother’s education level	0.629***	1					
	3.Ideal spouse’s masculinity	−0.030	−0.111**	1				
	4. Ideal spouse’s femininity	0.068	0.025	0.359***	1			
	5.Own masculinity	0.103**	0.082*	0.388***	0.220**	1		
	6. Own femininity	−0.046	−0.074	0.280***	0.569***	0.267***	1	
	7. Urban-rural	0.379***	0.417***	−0.028	0.076	0.158***	−0.078	1

*p < 0.05, **p < 0.01, ***p < 0.001.

#### Moderation model

Using the bootstrap estimation of moderation analysis, the father’s and mother’s education levels were the independent variables; the masculinity score of the ideal mate and femininity score of the ideal mate were the dependent variables; and urban-rural area was the proposed moderator. We transformed the urban-rural areas into a dummy variable (rural = 0, urban = 1). The results are shown in Table 5. Urban-rural area had a moderating effect on the relationship between the mother’s education level and the masculinity score of the ideal mate in males (*B* = 0.845, *p* = 0.011). The direct association between mothers’ education and sons’ ideal mate gender role was smaller for adolescents in rural areas than in urban areas. In females, urban-rural area had a moderating effect on the relationship between fathers’ education level and the femininity score of the ideal mate (*B* = −0.753, *p* = 0.007), and on the relationship between mothers’ education level and the femininity score (*B* = −0.791, *p* = 0.003). These associations were smaller for adolescents in urban areas than in rural areas.

**Table 5:** Urban-rural residence as a moderator of the association between parental education level and explicit mate preference.

	*X*	*Y*	*M* × *X* (*B*)	95% *CI*	
				Lower	Upper
Males	Father	Masculinity	0.389	−0.361	1.138
	Father	Femininity	0.630	−0.172	1.431
	Mother	Masculinity	0.845*	0.195	1.495
	Mother	Femininity	−0.138	−0.847	0.571
Females	Father	Masculinity	0.075	−0.553	0.702
	Father	Femininity	−0.753**	−1.303	−0.203
	Mother	Masculinity	−0.092	−0.682	0.498
	Mother	Femininity	−0.791**	−1.310	−0.271

*p < 0.05, **p < 0.01; *X*, education level; *Y*, gender role score of the ideal mate; *M*, urban-rural residence.

### Discussion

Consistent with previous studies, the results of the study show that the single gender role is no longer dominant (Juster et al., 2016; Lin et al., 2020), and the number of people who prefer the traditional single gender role is only approximately one-third of the total for mate preference. Together, these results indicate that people’s preferences for gender roles and mate choice in modern society are different from traditional roles.

If a mother with higher educational level, her male child (children) would be more likely to seek a mate with high-masculinity traits. If a father with higher educational level, his male child (children) would prefer a mate with high-masculinity and low-femininity traits. It is speculated that the reason behind this phenomenon is related to the social structure of contemporary China. In modern society, more and more women have abandoned the housewife role and entered the workforce, and women often need to display a degree of masculinity to be seen as successful in the workplace. For example, a study found that women who appeared more masculine were more likely to be team leaders than men (Lanaj and Hollenbeck, 2015). Bereczkei et al. (2002) believed that men internalize their mother’s phenotype as a template for acquiring similar mates, suggesting that the mother’s gender role characteristics affect men’s mate preference. Regarding the choice of career, the level of education is an important factor. Women with higher education are more likely to take non-traditional jobs. The mother’s personality may influence the formation of mate preferences during childhood. So their children’s mate preferences have higher score on non-traditional gender role characteristics.

In women, the higher the parents’ education level was, the more they preferred mates with a low degree of masculinity. This may reflect the influence of the social environment. In modern society, the jobs in which highly educated men engage are more likely to require feminine characteristics (Eagly and Karau, 1991; Roos and Manley, 1996; Park, 1997). For example, people who engage in high-level management work not only need masculine characteristics, such as leadership and judgment, but also feminine characteristics, such as considerateness and amiability, while people who engage in scientific research require high levels of carefulness. In addition, many studies found that individuals with high scores in both masculine and feminine dimensions are the most adaptable and psychologically healthy (Prakash et al., 2010; Stoker et al., 2012; Lin et al., 2021).

Urban-rural residence had a significant moderating effect on the association between parents’ education level and the students’ mate preferences. In male participants, the relationship between the mother’s education level and preference for masculinity in a mate was stronger in urban than in rural areas. It is possible that in rural areas, where work is more tradition with respect to gender roles than it is in urban areas, have fewer opportunities to work in non-traditional jobs, regardless of parents’ education level. In contrast, there is a greater range of jobs in urban areas, and the impact of parents’ education on their sons’ job placements may be more apparent. Parents with low education levels are more likely to work in jobs that match traditional gender roles, while parents with higher education are more likely to take jobs according to their abilities, rather than traditional gender roles. As a result, sons whose parents have high educational levels in urban areas are more likely to be exposed to an environment with non-traditional gender roles and more likely themselves to form different attitudes toward gender roles.

The findings were opposite for the female participants: the influence of parents’ education level on mate preference was stronger in rural areas than in urban areas. This may be because women in rural areas have a more traditional view of mate choice (Schvaneveldt and Hubler, 2012) and prefer less feminine. In contrast, women who are raised in a city may prefer men with more feminine traits as partners. This would mean that the women’s mate choices would be therefore less affected by the education level of the parents. Consistent with this possibility, Feng and Xiao (2014) found a significant gap between urban and rural areas in terms of female gender roles. Urban women are more likely to have non-traditional gender roles than rural women are, so men who display characteristics of non-traditional gender roles are more likely to be accepted by urban women.

## Study 2 implicit mate preference

### Methods

#### Participants

An effect size of *r* = 0.3 was used to conduct a power analysis with G*power 3.1 (Faul et al., 2007). The estimated sample size was no less than 82 to achieve 80% power to detect the effects given an α level of 0.05. A total of 130 Chinese heterosexual college students who were not involved in Study 1 volunteered to participate in the study (female = 66, age range = 19–26 years, mean age = 21.38 years).

#### Implicit measure of mate preference

The gender role of the ideal mate was assessed using an implicit measure. This type of measure may reveal attitudes that may not be apparent on self-report measures. The target term was “ideal mate.” The 16 masculinity words (e.g., brave, dominant) came from the masculinity subscales, and the 16 femininity words (e.g., soft, considerate) came from the femininity subscales. The administration of this implicit measure is described in the Procedure section.

#### Explicit measure of mate preference

Explicit measure of mate preference was assessed using the same method as in Study 1.

#### Urban-rural residence

Urban-rural residence was assessed using the same method as in Study 1.

#### Procedure

Presentation 0.71 software was used to run the experiment. The participants sat on a chair in a sound-insulated electronic-magnetic room. The screen was 80 cm away from the participants. The participants took the CSPI-50 before the experiment. We used a single-category implicit association test (SC-IAT; Karpinski and Steinman, 2006) to access the implicit attitude of mate preferences.

Two concepts and one target appear in a two-choice task. When the target shares the same response key with strongly associated concepts, response time is shorter than when the target shares the same response key with weakly associated concepts. This performance difference implicitly measures differential associations of the two concepts with the target. Compatible phase is when the target shares a key with strongly associated concepts. Incompatible phase is when the target shares a key with weakly associated concepts. In order to avoid the influences of task familiarity, the participants were required to carry out the practice phase before the formal phase. The experiment included four phases.

##### Compatible practice

The target words (“ideal mate”), masculinity words, and femininity words were presented in random order. The target word was presented 16 times. Each masculinity word was presented once. Each femininity word was also presented once. The female participants pressed the “F” key when the masculinity word or target word was presented and the “J” key when the femininity word was presented. The male participants pressed the “F” key when the masculinity word was presented and the “J” key when the femininity word or target word was presented. The order of the buttons was counterbalanced among the participants. Each word was displayed until the participant pressed the key. The screen displayed feedback (“right” or “wrong”) after the participant pressed the key. The ISI was 1,400 ± 200 ms.

##### Compatible formal

The 16 femininity words and 16 masculinity words were presented twice, and the target word (“ideal mate”) was presented 32 times. There was no feedback after pressing the key. The other procedures were the same as in the compatible practice phase.

##### Incompatible practice

The female participants pressed the “F” key when the masculinity word was presented and the “J” key when the femininity word or target word (“ideal mate”) was presented. The male participants pressed the “F” key when the masculinity word or target word was presented and the “J” key when the femininity word was presented. The other procedures were the same as in the compatible practice phase.

##### Incompatible formal

The femininity words and masculinity words were presented twice, and the target word (“ideal mate”) was presented 32 times. There was no feedback after pressing the key. The other procedures were the same as in the incompatible practice phase. All the participants completed the experiment in the order of (1) to (4).

#### Analysis

The analysis was performed using SPSS 24.0. Implicit priming is the reaction time to the target word of the incompatible formal items minus the compatible formal items. The lower the implicit priming is, the more likely a woman (man) is to prefer a mate with a lower masculine score and a higher femininity score (a higher masculine score and a lower femininity score) in implicit attitude.

### Results

#### Characteristics of participants

The characteristics of the participants are reported in Table 6.

**Table 6:** Characteristics of participants.

		*n*	*Mean*	*SD*
Age				
		130	21.39	1.97
Gender				
	Male	64		
	Female	66		
Urban-rural residence				
	Rural	51		
	Urban	79		
Education level				
	Father	130	10.59	3.36
	Mother	130	9.22	3.34
Gender role scores				
	Masculinity-ideal partner	130	84.95	11.43
	Femininity-ideal partner	130	86.10	11.62
Implicit priming (ms)				
		130	35.24	280.43

#### Correlations among parental education level, explicit mate preference, and implicit mate preference

Pearson’s correlations were used to analyze the associations among the study variables (Table 7). Among males, there was no correlation among the variables. Among females, the father’s education level (*r* = −0.309, *p* = 0.012) and mother’s education level (*r* = −0.248, *p* = 0.045) were negatively correlated with priming, suggesting that the higher the parent’s education level was, the more likely the female participant was to prefer a man with a lower masculine score and a higher femininity score. For both males and females, there was no correlation between explicit mate preference and implicit mate preference.

**Table 7:** Correlations between implicit mate preference and parents’ education level.

	Father’s education level	Mother’s education level	Masculinity	Femininity
Male’s priming	−0.094	−0.005	0.029	0.108
Female’s priming	−0.309*	−0.248*	−0.011	−0.084

*p < 0.05, **p < 0.01.

#### Moderation model

Using the bootstrap estimation of the moderation analysis, the father’s education level and mother’s education level were the independent variables; implicit priming (measured as reaction time) was the dependent variable; and the urban-rural area was the proposed moderator. The results are shown in Table 8. Urban-rural residence had no moderating effect on the relationship between the parents’ education level and implicit priming.

**Table 8:** Moderation model for implicit mate preference, parental education level and urban-rural areas.

	*X*	*M* × X (*B*)	95% *CI*	
			Lower	Upper
Males	Father	29.513	−28.126	87.151
	Mother	−53.353	−113.297	6.590
Females	Father	−19.307	−58.816	20.202
	Mother	−22.413	−61.353	16.527

*X*, education level; *M*, urban-rural residence.

### Discussion

Consistent with previous research results (Huang, 2013), there was a difference between implicit and explicit attitudes regarding mate preference, suggesting that explicit mate preference may be influenced by the effect of social desirability or other assumptions about social behavior. Among female participants, the parents’ education level was negatively correlated with the implicit attitudes about mate preference, and this association was not moderated by urban-rural residence. Specifically, the higher the parents’ education level was, the more likely the female participants were to accept male mates with non-traditional gender role characteristics, regardless of area of residence. In contrast, among male participants, there was no significant correlation between the parents’ education level and implicit mate preference, and nonsignificant correlation was observed regardless of area of residence.

The IAT is less influenced by conscious awareness than self-report measures are. The results suggested that urban-rural residence only influenced mate preferences at the conscious level. Similarly, parents’ education level was associated only with women’s mate preferences, not men’s preferences. Women may be more likely to be influenced by their parents, while men may be more independent in their mate preferences.

## General discussion

The paper examined the influence of parental education level on mate preference in two studies, and the results showed that parents’ education level was significantly correlated with their children’s mate preference. In male participants, the higher the parents’ education level was, the more men preferred female partners with a higher degree of masculine traits and a lower degree of feminine traits. In female participants, the higher the parents’ education level was, the more women preferred male partners with a lower degree of masculine traits and a higher degree of feminine traits. This is the first study to test the relationship between parents’ education level and the gender role characteristics of college students’ preferences for an ideal mate.

Parents’ education level was associated with both explicit and implicit measures of mate preference, which may be because parental influence occurs in the early stages of individuals’ growth rather than being instilled by others later. Thus, implicit attitudes can also be affected. Parents with different education levels have different parenting styles, parent–child relationships, living environments, relatives, and friends than parents with lower education (Zhang et al., 2015). According to the ecological systems theory (Bronfenbrenner, 1986), microsystems, mesosystems, and exosystems simultaneously influence the formation of individual ideas until the individual becomes an adult with stable values. The parents’ education level greatly affects the composition of these systems. Thus, the whole ecosystem of children’s growth includes large differences, which allows the individual’s implicit preference for mate selection to have significant differences.

Furthermore, the influence of the parents’ education level was stronger on the implicit mate preference of women than men. This may be due to the higher requirement of feminization among males than to the masculinization of females in modern society. For example, many jobs in which males engage require feminine characteristics (Eagly and Karau, 1991; Roos and Manley, 1996; Park, 1997). Some studies found that females express a preference for feminized over masculinized male faces (Perrett et al., 1998; Welling et al., 2009; Burriss et al., 2014). Additionally, there was a strong correlation between gender role attitudes and domestic violence. People who preferred traditional gender roles attitudes were more supportive of violence against women (Berkel et al., 2004). Females were more likely to prefer more feminized males given the advantages of femininity in the work, mate selection, and family.

A meta-analysis conducted by Greenwald et al. (2009) found a weak correlation between explicit and implicit measures. Self-report results would be affected by social desirability. As mentioned above, there were significant differences in mate preference between urban and rural areas (Schvaneveldt and Hubler, 2012; Prakash and Singh, 2014). In study 1, the moderating effect of urban-rural may be derived from the social desirability of gender roles. While in study 2, the results of IAT were more automatic and less susceptible to biases and other distortions (Zayas and Shoda, 2016). As a result, there were differences in the moderating effect of urban-rural between study 1 and study 2.

With societal changes, there have also been changes in the abilities required by various professions and are significantly different from the past. As a result, both the modern career needs for non-traditional gender characteristics and the advantages of non-traditional gender characteristics have an important influence on mate preference. The results showed that the parents’ education level not only affects explicit mate preference but also affects implicit attitude. It is believed that with increases in education level in China associated with modernization, the advantages of androgyny will become increasingly obvious.

## Limitations and future research

The current studies revealed a fascinating fact that parents’ education level is associated with the gender role of an ideal mate for their children, but the results need to be considered prudently due to the following aspects: First, we only recruited younger adults from college, whose attitudes toward ideal partner are likely to be influenced by their families, especially parents. However, we are not sure whether their attitudes change as they graduate or grow older, and further studies referring to a broader social sample are needed. Second, many studies have found cultural differences in mate preferences (Mafra et al., 2020; Thomas et al., 2020). The results of the present study may not be applicable to different cultural backgrounds. A cross-culture study will help to understand these differences. Third, the findings from correlation analysis should be interpreted with caution because of the limitations of zero-order intercorrelations without control of multiple testing.

## Data availability statement

The original contributions presented in the study are included in the article/Supplementary material, further inquiries can be directed to the corresponding author.

## Ethics statement

The studies involving human participants were reviewed and approved by the Institute of Brain and Psychological Science, Sichuan Normal University (ID: SCNU-201015). The patients/participants provided their written informed consent to participate in this study.

## Author contributions

WL and JL contributed to conception and design of the study. WL, JW, and ZL organized the database. WL performed the statistical analysis. WL wrote the first draft of the manuscript. WL, JL, YL, and JW wrote sections of the manuscript. All authors contributed to manuscript revision, read, and approved the submitted version.

## Funding

This study was supported by grants from the Key Projects of Philosophy and Social Sciences Research, Ministry of Education [21JZD063] and Guangdong Basic and Applied Basic Research Foundation [2021A1515011259].

## Conflict of interest

The authors declare that the research was conducted in the absence of any commercial or financial relationships that could be construed as a potential conflict of interest.

## Publisher’s note

All claims expressed in this article are solely those of the authors and do not necessarily represent those of their affiliated organizations, or those of the publisher, the editors and the reviewers. Any product that may be evaluated in this article, or claim that may be made by its manufacturer, is not guaranteed or endorsed by the publisher.

## Footnotes

^1^ www.wjx.cn
