# Supplementary material for: The relationship between Chinese college students’ mate preferences and their parents’ education level
Source: Front Psychol. 2022 Nov 1;13:907315. doi: 10.3389/fpsyg.2022.907315 (PMC9664191; doi:10.3389/fpsyg.2022.907315)
Supplement: Supplementary file 1 [file Table_1.DOCX]

a001-a016 is the masculinity words of practice phase

b001-b016 is the femininity words of practice phase

c001 is the target term of practice phase

a101-a116 is the masculinity words of incompatible formal phase for male participant (compatible formal phase for female participant)

b101-b116 is the femininity words of incompatible formal phase for male participant (compatible formal phase for female participant)

c101 is the target term of incompatible formal phase for male participant (compatible formal phase for female participant)

a201-a216 is the masculinity words of compatible formal phase for male participant (incompatible formal phase for female participant)

b201-b216 is the femininity words of compatible formal phase for male participant (incompatible formal phase for female participant)

c201 is the target term of compatible formal phase for male participant (incompatible formal phase for female participant)
